# Supplementary material for: Neonatal Hypoxic-Ischemic Brain Injury Alters Brain Acylcarnitine Levels in a Mouse Model
Source: Metabolites. 2022 May 22;12(5):467. doi: 10.3390/metabo12050467 (PMC9143624; doi:10.3390/metabo12050467)
Supplement: Supplementary file 1 [file metabolites-12-00467-s001.zip › Supplemental Figure 1,2 Acylcarnitine 32022.pdf]

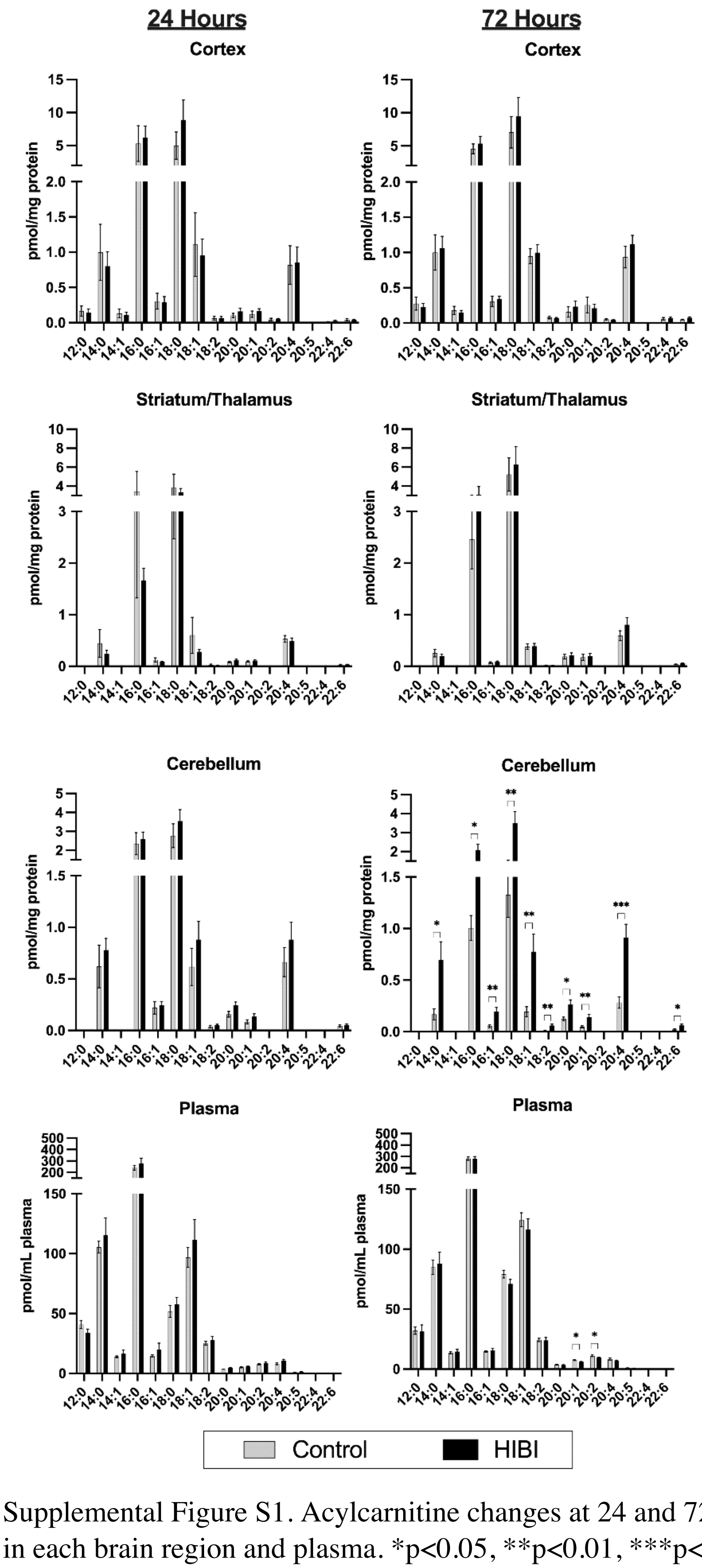

Supplemental Figure S1. Acylcarnitine changes at 24 and 72 hours after HIBI versus controls in each brain region and plasma. *p<0.05, **p<0.01, ***p<0.001, ****p<0.0001.

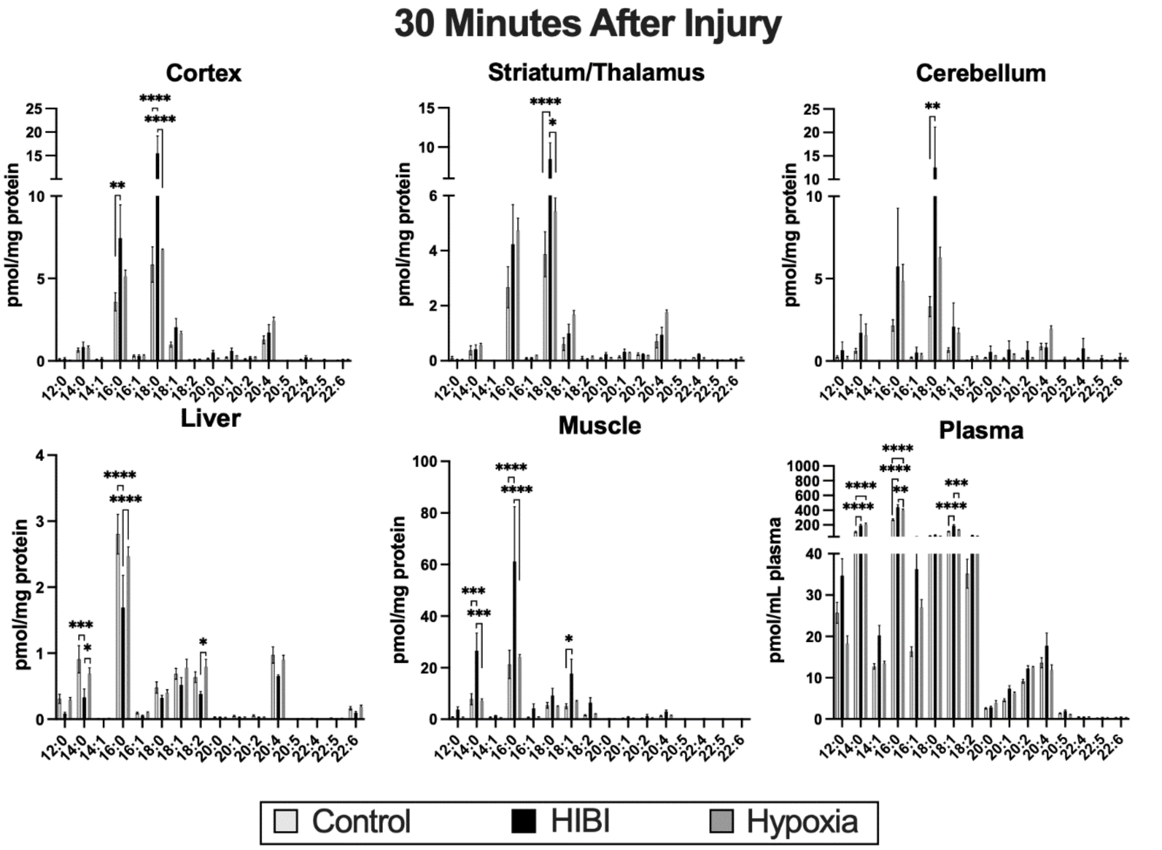

Supplemental Figure S2. All acylcarnitine species levels in brain regions, liver, muscle, and plasma under all three conditions. *p<0.05, **p<0.01, ***p<0.001, ****p<0.0001.
