# Supplementary figures and images for: Neonatal Hypoxic-Ischemic Brain Injury Alters Brain Acylcarnitine Levels in a Mouse Model
Source: Metabolites. 2022 May 22;12(5):467. doi: 10.3390/metabo12050467 (PMC9143624; doi:10.3390/metabo12050467)

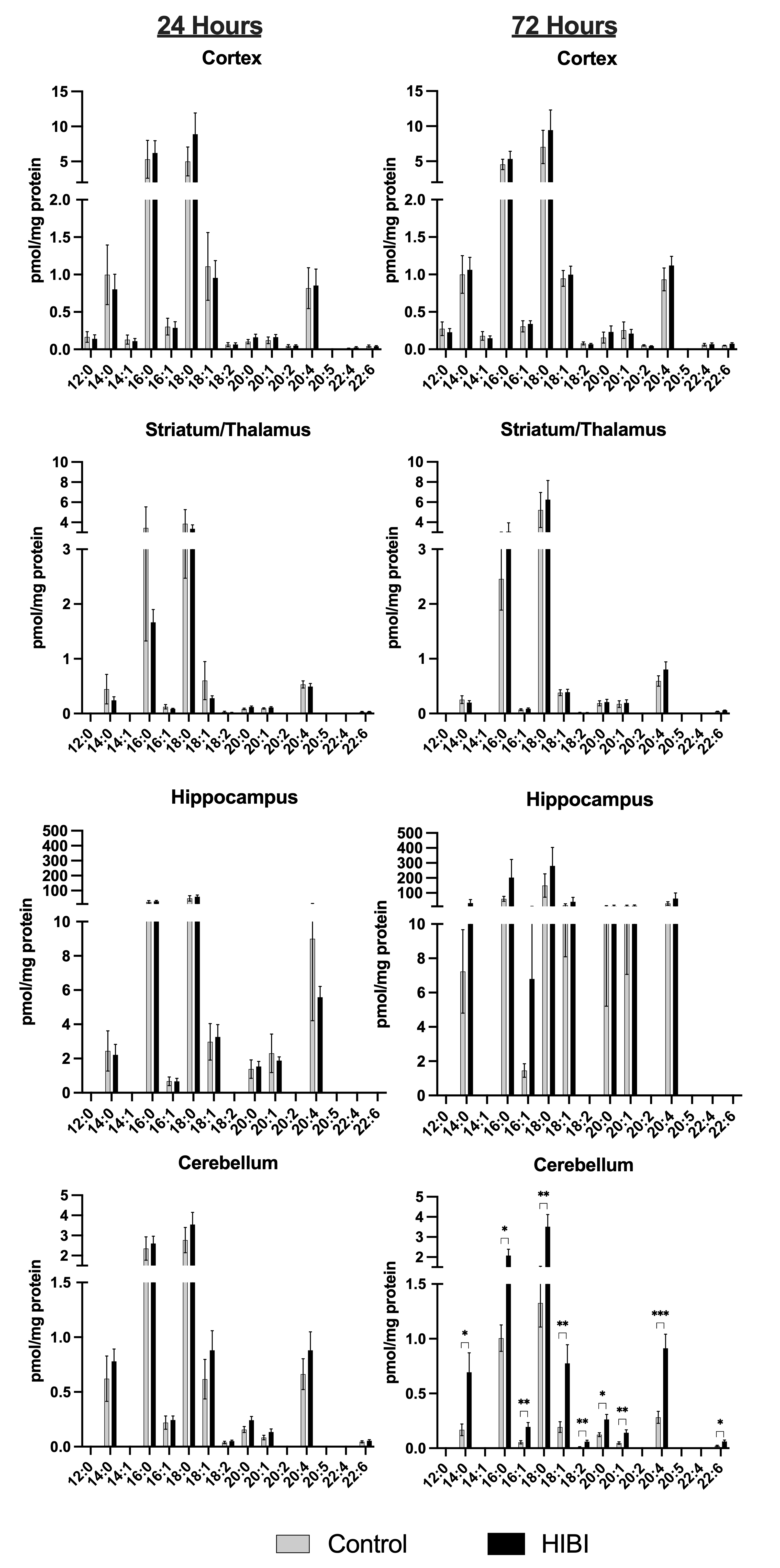

Supplement: Supplementary file 1 [file metabolites-12-00467-s001.zip › Supp Figure S1.tiff]
